# Characterization, Genomic Analysis and Application of Five Lytic Phages Against Carbapenem-Resistant *Pseudomonas aeruginosa*

**DOI:** 10.3390/microorganisms13071587

**Published:** 2025-07-05

**Authors:** Li-Ping Zhang, Chang-An Li, Yongda Zhao, Zeqing Wang, Junjie Wang, Feng-Jing Song, Bao-Tao Liu

**Affiliations:** 1 College of Veterinary Medicine, Qingdao Agricultural University, Qingdao 266109, China; 200601172@qau.edu.cn (L.-P.Z.); changan_522@163.com (C.-A.L.); wangzeq521@gmail.com (Z.W.); 16634254313@163.com (J.W.); 2 Qingdao Bolin Biotechnology Co., Ltd., Qingdao 266000, China; ydzhao_house@126.com; 3 Institute of Plant Protection, Qingdao Academy of Agricultural Sciences, Qingdao 266100, China

**Keywords:** characterization, genomic analysis, *Pseudomonas aeruginosa*, carbapenem resistance, lytic phage

## Abstract

The high pathogenicity rate of carbapenem-resistant *Pseudomonas aeruginosa* (CRPA) has resulted in substantial economic losses for humans and the breeding industry. Consequently, there is an urgent need to develop new alternatives to mitigate antibiotic use. Phage therapy has demonstrated promising results in numerous studies. In this study, lytic phages targeting CRPA were isolated from feces and river water samples in Shandong, China. A total of 94 phage strains with CRPA as hosts were obtained, exhibiting lysis rates that ranged from 29% to 76% for *P. aeruginosa* derived from humans and different types of animals (n = 246). We further examined five representative phages, the host bacteria of which were CRPA from clinical patients and poultry, and these phages included two myoviruses and three podoviruses. Their optimal multiplicities of infection (MOIs) ranged from 10^−3^ to 10^−5^, with latent periods of less than 5 to 15 min and burst durations of 140 to 175 min, resulting in burst sizes of 133 to 352 PFU/cell. All five phages exhibited the ability to survive at temperatures up to 60 °C and within pH levels of 3 to 11. Whole-genome sequencing revealed that these five phages were all double-stranded DNA phages and did not possess resistance genes or virulence factors. The two myoviruses, sharing similar sequences, were classified into the genus *Pakpunavirus*, with a size of 92,509 bp and 92,293 bp, 149 to 152 ORFs and 20 to 22 tRNAs. In contrast, the three similar podoviruses belong to the genus *Phikmvvirus* and all contained a perforin–lyase system, with a size of 43.35 kb, a GC content of 62%, 49 to 50 ORFs and 16 to 20 tRNAs. A spray disinfection experiment demonstrated that the phage cocktail exhibited a high sterilization effect after spraying and showed good efficacy against cement and metal surfaces. This study provides foundational information for further research into the elimination of CRPA in the environment.

## 1. Introduction

*Pseudomonas aeruginosa* is an opportunistic Gram-negative pathogen commonly found in soil and water as well as in poultry, livestock, wildlife, food and the environment [1]. The inappropriate use of antibiotics has enabled *P. aeruginosa* to develop significant resistance to a variety of antibiotics, leading to high disease incidence and mortality in both livestock and humans [2]. Carbapenems are considered the first choice for the treatment of multidrug-resistant *P. aeruginosa* in humans [3]. However, the increasing use of beta-lactams including carbapenems has resulted in the emergence and rapid spread of carbapenem-resistant *P. aeruginosa* (CRPA) around the world, posing a threat to human health and safety [4]. In recent years, the prevalence of CRPA in clinical settings has increased significantly. A susceptibility survey of 1127 strains of *P. aeruginosa* in Taiwan showed that the annual non-susceptibility rate of *P. aeruginosa* to carbapenem antibiotics increased from 19.7% in 2016 to 27.5% in 2018 [5]. In 2012, migratory birds in southern Brazil were monitored for Gram-negative bacilli, and 9 CRPA strains were found among 300 strains of Gram-negative bacteria [6]. Therefore, there is an urgent need for effective alternatives or supplemental treatments including phage therapy to reduce antibiotic use [7,8,9].

Phages targeting *P. aeruginosa* were first discovered in the mid-20th century. *P. aeruginosa* is highly resistant to antibiotics and commonly found in hospital infections. Therefore, the use of phages to inhibit *P. aeruginosa* has received great attention [10]. In particular, the application of phage cocktail therapy (a mixture of two or more phages) is typically more effective in inhibiting bacterial infections than a single phage [11]. Phage cocktails in vitro can effectively reduce infections caused by *P. aeruginosa* and have shown positive effects in clinical trials [12]. For example, a therapeutic phage preparation targeting multidrug-resistant *P. aeruginosa* infections was examined in 12 patients with chronic otitis media, and the patients showed significant clinical improvement at 7, 21 and 42 d following the treatment with the single-dose phage preparation [13]. Phages have an advantage over antibiotics in inhibiting infections caused by bacterial biofilms, since phages can penetrate biofilm inner layers that antibiotics struggle to. Phages can eliminate *P. aeruginosa* biofilms formed by isolates from patients with chronic sinusitis [14]. Phages can also be genetically modified to induce the synthesis of quorum-quenching lactamases that inhibit biofilms by hydrolyzing acyl-homoserine lactones and suppressing quorum-sensing activity [15]. In addition, compared to antibiotics, phages have a strong specificity for their host bacteria and can reproduce in large numbers in a short period to lysogenize bacteria, but they can be naturally metabolized by the body [16,17]. Therefore, phage cocktails are considered a safe and effective treatment. Surface disinfection is a key measure to control *P. aeruginosa* spread, as this pathogen can survive for a long time on nonbiological surfaces such as medical equipment, floors and metal devices, causing cross-contamination in medical and agricultural environments. Research has shown that the environmental host of *P. aeruginosa* has a significant impact on hospital outbreaks and the spread of zoonotic diseases [18]. Animals and animal products may serve as a primary source of *Pseudomonas* spp., which can be potentially transmitted to humans [19]. However, there are very few studies about phages targeting multidrug-resistant (MDR) *P. aeruginosa*, especially CRPA, from both animals and patients. It is necessary to obtain phages which could lyse MDR *P. aeruginosa* from both animals and humans.

In this study, CRPA phages were isolated from different sources in Shandong, and five representative phages were selected to further analyze their biological and genomic characteristics. Our obtained phages could not only lyse CRPA from different animal sources but also cleave CRPA from patients. The phage cocktail exhibited a high sterilization effect against cement and metal surfaces. The representative phages have the potential to be used as antibacterial agents in animal production and in humans, as well as disinfectant to control CRPA transmission.

## 2. Materials and Methods

### 2.1. Bacteria Strains

A total of 246 *P. aeruginosa* strains including 73 meropenem-resistant *P. aeruginosa* strains were previously identified by PCR and kept in the Veterinary Pharmacology Lab of Qingdao Agricultural University. The 246 *P. aeruginosa* strains included 30 from ducks, 55 from laying hens, 49 from broilers, 44 from pigs, 45 from geese and 23 CRPA strains from patients. All strains were cultured in Luria–Bertani (LB) medium at 37 °C.

### 2.2. Sample Sources and Processing

A total of 122 samples were collected from animal farms and rivers in Shandong, China, between 2020 and 2022 for the purpose of phage isolation. The samples included 36 from feces and 86 from river water. To isolate broad-spectrum phages targeting isolates from different sources, nine CRPA strains of various sources were randomly selected as host bacteria, including one from a duck (CD5-6-2), one from a broiler (SE-C13-1), three from layer chickens (SE-134, DS-C44-1 and DS-C59-1), one from a pig (SE-P45-2), one from a goose (G1-7-1) and two from clinical patients (CR-PA20026945 and CR-PA20025438).

For the feces samples, each sample of about 15 g was mixed with 15 mL SM (Sodium chloride/Magnesium sulfate) buffer and then centrifuged at 10,000 rpm for 10 min. The supernatant was then filtered through a 0.22 μm membrane to ensure sterilization. The river water was centrifuged at 10,000 rpm for 10 min, followed by filtration through a 0.22 μm filter. The obtained filtrate was stored at 4 °C for future use.

### 2.3. Phage Isolation and Purification

Phages were isolated and purified using the double-layer agar method described previously [20]. The filtrate (10 mL) was mixed with all eight host bacteria (200 µL each, 10^9^ CFU/mL) in 100 mL of LB broth and incubated at 37 °C overnight to enrich phages, followed by centrifugation at 10,000 r/min for 10 min at 4 °C. The supernatant was filtered through a sterile 0.22 μm membrane. The steps for enriching phages were repeated three times. The host bacteria (200 μL) were inoculated into LB broth (10 mL) and cultured to the logarithmic phase. The mixture of bacteria and filtrate was mixed with 5 mL LB agar (0.7%) thoroughly and poured onto the top of LB plates, followed by incubation at 37 °C for 12 h. Phage plagues were purified by the double-layer agar method as previously described with minor modifications [20]. In brief, the clear plaques on the plates were picked and placed in 1 mL SM buffer (100 mM NaCl, 8 mM MgSO_4_, 50 mM Tris-HCl, pH 7.5) overnight at 4 °C to detach the phage particles from the agar. After filtering through a 0.22 μm filter, 100 μL of the filtrate was diluted serially and each dilution (100 μL) was mixed with the corresponding host bacteria. The mixture (200 μL) of bacteriophages and host bacteria was added into 5 mL of LB agar (0.7%) and quickly poured onto LB agar plates. The plates were incubated overnight at 37 °C to obtain individual plaques. The purification described above was repeated at least three times until the shape and size of the plaque were consistent. The phages were preserved in glycerol at −80 °C.

### 2.4. Phage Lysis Rate

The lysis rates of phages were determined by spot assays and verified in classic whole-plate plaque assays, which used the double-layer agar method [21]. Briefly, logarithmic-phase bacterial cultures (100 μL, ~10^8^ CFU/mL) were mixed with 5 mL of LB agar (0.7% agar) and overlaid onto LB agar plates. Serial 10-fold dilutions (10^0^–10^−8^) of phages were prepared in SM buffer and spotted onto the bacterial lawn (n = 3 replicates per phage). After overnight incubation at 37 °C, plaque formation was assessed. Bacteria with clear plaques were scored as susceptible and those with no plaques were scored as resistant. The phage lysis rate was defined as the spectrum of bacterial cells that a phage could infect and lyse [22]. The lysis rate (%) was calculated as follows: lysis rate (%) = (number of lysed strains/tested strains) × 100%. Three independent experiments were performed to ensure the accuracy of the results.

### 2.5. Morphological Characterization

Five representative phages with lysis rates ≥ 46.5% against 73 CRPA strains were selected, and their morphology was observed using a transmission electron microscope (HT7700, Hitachi, Tokyo, Japan) as previously described [23]. Briefly, the phage suspensions (≥10^9^ PFU/mL) of 20 μL were dropped on the carbon-coated grids, adsorbed for 10 min and then stained with 2% uranyl acetate for 5 min in darkness. The grids were then air-dried at 70 °C in the dark, and morphological characterization of the phages was conducted using the TEM (Hitachi, Japan) at an accelerated voltage of 80 kV.

### 2.6. Optimal Multiplicity of Infection (MOI) and One-Step Growth Curve

The optimal MOI of each representative phage was determined as previously described with minor modifications [24]. In brief, the phage titer was measured using the double-layer agar plate method. The phage and host strain (10^8^ CFU/mL) were mixed at different ratios (10, 1, 0.1, 0.01, 0.001) and co-cultured at 37 °C for 4 h. The phage titer was assessed using the double-layer agar method to determine the optimal MOI. A one-step growth curve was established as previously described [25]. In brief, phage titers were adjusted to MOI = 0.01 using bacteria in the logarithmic growth phase that were incubated at 37 °C for 10 min, followed by centrifugation at 10,000 rpm for 1 min. The precipitate was washed twice with LB broth and then resuspended in 1 mL LB broth, followed by incubation at 37 °C with shaking. Samples of 20 μL were collected at 0, 5, 10, 15, 20, 25, 30, 40, 50, 60, 70, 80, 90, 110, 130, 150, 180 and 210 min. These samples were centrifuged at 10,000 rpm for 1 min, and supernatants were filtered through 0.22 μm membranes and then serially diluted 10-fold. The burst size was determined using the ratio of the final number of released phage particles to the initial count of phage particles [26]. All these experiments were repeated in triplicate.

### 2.7. Thermal Stability and pH Tolerance

For thermal stability, phage suspensions were incubated at 40, 50, 60, 70 and 80 °C and then sampled for incubation periods of 20, 40, 60 and 80 min for titer analysis. For pH tolerance, the bacteriophage suspensions were adjusted to different pH values (2, 3, 4, 5, 6, 7, 8, 9, 10, 11, 12, 13) and incubated at 37 °C for 2 h. After serial 10-fold dilutions, the phage titer was determined using the double-layer plate method [27]. These experiments were conducted in triplicate.

### 2.8. Sequencing and Genome Analysis

The genomic DNA of the five representative phages was extracted using a modified phenol–chloroform method as previously described [28]. Briefly, the DNA was extracted by adding phenol–chloroform–isoamyl alcohol (25:24:1) to the phage suspension which was treated with DNase I and RNase A. After centrifugation at 10,000 g for 10 min at 4 °C, the aqueous layer was collected and mixed with isopropanol, followed by maintenance at –20 °C for 4 h. After centrifugation at 10,000 g for 30 min at 4 °C, the DNA pellets were washed with 75% ethanol three times and then resuspended in nuclease-free water. The DNA was quantified with the Nanodrop (Agilent 5400, Agilent Technologies, Inc., Santa Clara, CA, USA) and then sent to Guangdong magigene Technology company for sequencing using the NexteraXT (Illumina, San Diego, CA, USA). The software Soapnuke (v2.0.5) was used to assess the quality of the sequencing data and remove low-quality data. BWA (v0.7.17) software was used to map the clean reads to the *P. aeruginosa* genome, removing the host bacterial sequences. Assembly software Megahit (v1.1.2) was used to assemble the high-quality reads of each phage to obtain the contig sequences, and MetaGeneMark was used to predict the gene sequences of the phages. Resfinder 4.7 (http://genepi.food.dtu.dk/resfinder)(accessed on 2 June 2024), VirulenceFinder 2.0 (https://cge.food.dtu.dk/services/VirulenceFinder/) (accessed on 3 June 2024) and VFDB (http://www.mgc.ac.cn/cgi-bin/VFs/v5/main.cgi) (accessed on 3 June 2024) were used to identify whether there were antibiotic resistance genes or virulence genes. The RAST website (https://rast.nmpdr.org/) (accessed on 4 June 2024) was used to annotate phage genomes. BLAST 2.13.0+ (https://blast.ncbi.nlm.nih.gov/Blast.cgi) (accessed on 4 June 2024) was used to find highly similar phage genomes. The phylogenetic tree of phages was constructed using MEGA 6.0 software, and comparative analysis of the five phages was performed using BRIG version 0.95.

### 2.9. Cocktail Spray Disinfection Effect

In order to assess the effect of the phage cocktail on mixed contamination with pathogens which were not the host bacteria (Table S1), a spray disinfection experiment was conducted as previously described with minor modifications [29]. Briefly, each of the five representative phages was adjusted to three concentrations (10^3^, 10^6^, 10^9^ PFU/mL). Then, phage cocktails at the three concentrations (10^3^, 10^6^, 10^9^ PFU/mL) were made by equally mixing the five phages at the three concentrations (10^3^, 10^6^, 10^9^ PFU/mL). The inside of sealed sterile transparent boxes was continuously sprayed with a humidifier using the three cocktail reagents. Six CRPA strains (duck strain CD4-4-1, broiler strain SE-C44-1, layer strain JMA210PA, pig strain SE-P45-2, goose strain G1-7-1, and patient strain CR-PA20026836) which were not the host bacteria of the phages were mixed in equal proportion to prepare a bacterial solution at a concentration of 10^5^ CFU/mL. The bacterial mixture solution (100 μL) was spread onto LB agar plates, which were open and placed into the sterile transparent boxes with the humidifier. At 1, 3 and 6 h post-spray, three plates of each experiment group were selected for incubation at 37 °C for 16 h. PBS solution without a phage was used as the control group. The efficacy of the phage cocktail disinfection was assessed based on the average number of colonies on the plates.

### 2.10. Disinfection Effect on Cement and Metal Surfaces

Because cement floors and metal door handles might be dissemination media for resistant bacteria, which has a significant impact on hospital outbreaks and the spread of zoonotic diseases, cement and metal surface disinfection was also performed. Briefly, two CRPA strains (patient strain CR-PA20026836 and layer strain JMA210PA) were adjusted to 10^6^ CFU/mL and mixed at a 1:1 ratio. One milliliter (1 mL) of the bacterial suspension was transferred to a sterile spray bottle. The preparation (10^9^ PFU/mL) of phage vB_PaeP_QSZH was placed into a sterile spray bottle for spray application.

A transparent storage box (disinfected with 75% ethanol) containing two sterilized cement blocks was used to simulate a *P. aeruginosa*-contaminated environment. The bacterial suspension was evenly sprayed onto the blocks, followed by 2 mL of phage spray. After sealing the box for 30 min to allow phage sedimentation, a negative control (sterilized block sprayed with PBS) was processed in parallel. Surface-disinfected blocks were rinsed with 5 mL PBS for 5 min, and the eluent was serially diluted for plating on cetrimide agar (Hopebio, Qingdao, China). Following 24 h incubation at 37 °C, disinfection efficacy was quantified by comparing bacterial concentrations in PBS rinsates (CFU/mL) between treated and control groups. The experiments were performed in triplicate.

Door metal handles were disinfected with 75% ethanol and were divided into two groups. Each handle in Group 1 and 2 received 1 mL of CRPA suspension (10^6^ CFU/mL), and 5 min later, handles in Group 1 and 2 were treated with 1 mL phage spray (10^9^ CFU/mL) and PBS, respectively. After 30 min of exposure, each handle was wiped with sterile gauze moistened with PBS for 30 s (including mechanical pressure and movement path). The swab was then vortexed in 5 mL PBS for 30 s to fully wash out the bacteria, and the eluent was diluted in a series and spread onto cetrimide agar. All elution volumes, vortex times and dilution steps were strictly standardized to eliminate the impact of operational differences on bacterial counts. After incubating at 37 °C for 20 h, the disinfection effect was quantified by comparing the CRPA concentration (CFU/mL) in PBS eluents from different groups.

### 2.11. Statistical Analysis

All experiments were conducted in triplicate. All measurements were visualized using GraphPad Prism 8.0. Unpaired t-tests were used to evaluate the effects of phage treatments. Significance was set at *p* < 0.05, and the threshold for highly significant trends was set at *p* < 0.01.

### 2.12. Nucleotide Sequence Accession Numbers

Whole-genome sequences for the representative phages in this study were deposited in the GenBank database, and the accession numbers are PV752213-PV752216.

## 3. Results

### 3.1. Phage Isolation and Identification

A total of 94 CRPA phages were isolated from animal feces and river water in Shandong Province, China. To evaluate their host range, these phages were tested against 246 *P. aeruginosa* strains (73 CRPA and 173 meropenem-non-resistant strains). The 73 CRPA strains were from ducks (6 strains), broilers (11 strains), laying hens (21 strains), pigs (5 strains), geese (7 strains) and humans (23 strains). Among the 94 phages, 72 possessed lysis rates above 40.0% against the 246 *P. aeruginosa* strains and 13 showed lysis rates above 50.0% (Table 1). Notably, the five CRPA phages (vB_PaeM_QDB, vB_PaeM_ZGC, vB_PaeP_YZ2, vB_PaeP_QSZH and vB_PaeP_YQZQ,) from different sources showed high lysis rates, ranging from 46.5% to 63.0%, against the 73 CRPA strains, including the 23 human clinical CRPA strains (Table 1); thus, they were selected for further research. The host bacteria of the above five phages were CRPA strains CR-PA20026945 from a patient, DS-C44-1 from a layer, SE-C134 from a broiler, and CR-PA20025438 and CR-PA20025438 from patients. After incubation at 37 °C for 12 h on the double-layer agar plates, all five CRPA phages formed a clear round plaque, and the diameter of the plaque was about 3 mm, 2 mm, 7 mm, 7 mm and 7 mm, respectively (Figure S1). In particular, vB_PaeP_YZ2, vB_PaeP_QSZH and vB_PaeP_YQZQ exhibited a clear round plaque at the center, surrounded by a fuzzy halo.

### 3.2. Phage Electron Microscopy

**Figure 1:**
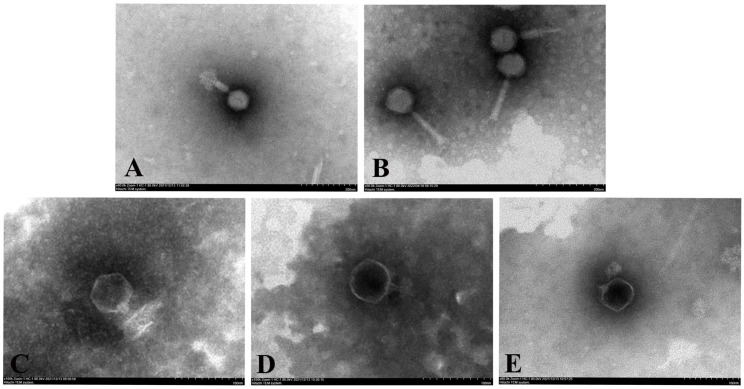
Morphology of the five phages under transmission electron microscopy. vB_PaeM_QDB (**A**), vB_PaeM_ZGC (**B**), vB_PaeP_YZ2 (**C**), vB_PaeP_QSZH (**D**) and vB_PaeP_YQZQ (**E**).

TEM images showed that phages vB_PaeM_QDB and vB_PaeM_ZGC exhibited regular icosahedral heads of 80 and 70 nm in diameter and the tail lengths were 125 and 120 nm, respectively (Figure 1). These were classified as order *Caudovirales*, family *Pakpunavirus*. In contrast, vB_PaeP_YZ2, vB_PaeP_QSZH and vB_PaeP_YQZQ, featured regular icosahedral heads with head lengths of 60, 62 and 60 nm, respectively, and their tail lengths were all 15 nm. These were classified as order *Caudovirales*, family *Phikmvvirus*.

### 3.3. Phage Biological Characteristics

#### 3.3.1. Optimal MOI and the One-Step Growth Curve

**Figure 2:**
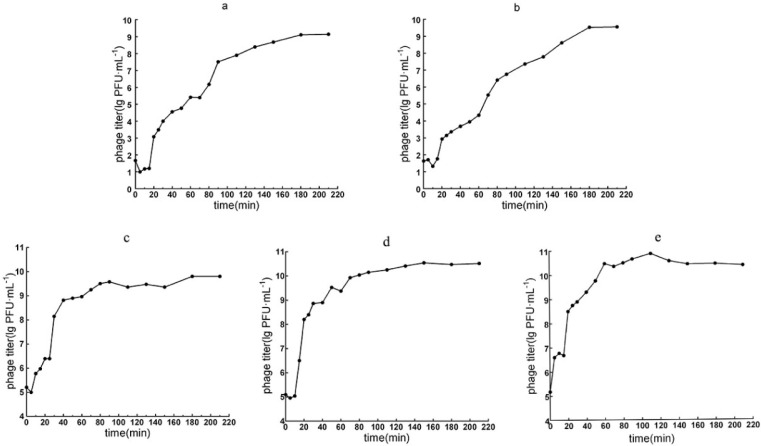
One-step growth curves for phages vB_PaeM_QDB (**a**), vB_PaeM_ZGC (**b**), vB_PaeP_YZ2 (**c**), vB_PaeP_QSZH (**d**) and vB_PaeP_YQZQ (**e**).

At MOIs of 0.0001 to 0.001, the phage titer reached the highest value of ~10^9^~10^12^ PFU/mL, indicating that the optimal MOIs of the five phages were 0.0001 to 0.001. The one-step growth curves indicated similar latent (15 min) and lytic burst periods (165 min) for the two *Pakpunavirus* phages vB_PaeM_QDB and vB_PaeM_ZGC. Phage titers ceased to increase around 180 min postinfection, leading to a stationary phase characterized by a burst size of approximately 140 and 133 PFU/cell, respectively (Figure 2a,b).

The one-step growth curves for the three *Phikmvvirus* phages vB_PaeP_YZ2, vB_PaeP_QSZH and vB_PaeP_YQZQ exhibited latent periods of 5, 10 and <5 min followed by lytic burst phases lasting 175, 140 and 140 min, respectively. The stationary phase was reached at 180, 150 and 145 min postinfection and exhibited burst sizes of 265, 352 and 266 PFU/cell, respectively (Figure 2c–e).

#### 3.3.2. Phage Temperature Tolerance

**Figure 3:**
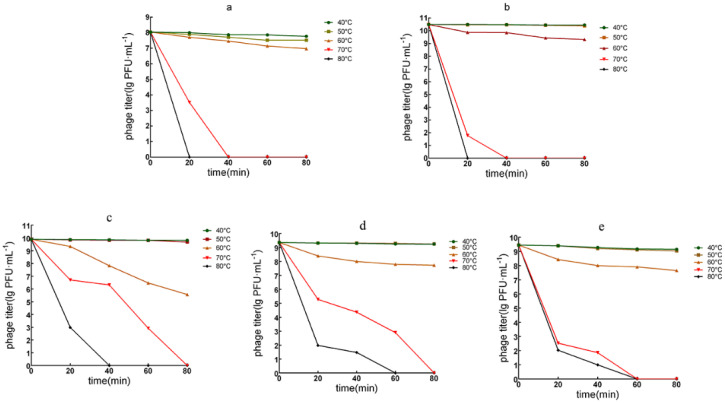
Temperature tolerance of the five phages in this study. (**a**) vB_PaeM_QDB stability against thermal stress; (**b**) vB_PaeM_ZGC stability against thermal stress; (**c**) vB_PaeP_YZ2 stability against thermal stress; (**d**) vB_PaeP_QSZH stability against thermal stress; (**e**) vB_PaeP_YQZQ stability against thermal stress.

The temperature tolerance of the two myoviruses, vB_PaeM_QDB and vB_PaeM_ZGC, exhibited high potency after 80 min of treatment at 40 and 50 °C. However, their effectiveness decreased by 11–13% following 80 min of exposure at 60 °C, and there was complete inactivation after 20 min at 80 °C. Notably, the temperature tolerance of vB_PaeM_QDB was superior to that of phage vB_PaeM_ZGC, because the potency reduced by 54% after 20 min at 70 °C, while there was a 73% reduction for vB_PaeM_ZGC under the same conditions (Figure 3a,b).

The tolerance for the three podoviruses indicated that they all (vB_PaeP_YZ2, vB_PaeP_QSZH and vB_PaeP_YQZQ) remained potent after 80 min at 40 and 50 °C. In contrast, their potency decreased significantly after 20 min at 70 °C with reductions of 70, 79 and 78%, respectively. At 80 °C, phage vB_PaeP_YZ2 was inactivated after 40 min, while the remaining two were inactivated after 60 min at this temperature (Figure 3c–e).

#### 3.3.3. pH Stability

**Figure 4:**
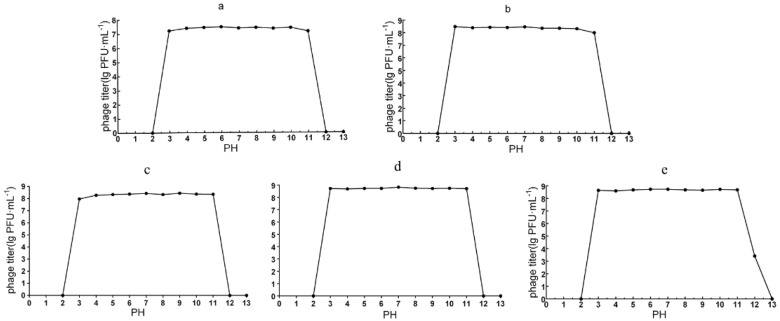
PH tolerance of the five phages. (**a**) vB_PaeM_QDB stability against pH stress; (**b**) vB_PaeM_ZGC stability against pH stress; (**c**) vB_PaeP_YZ2 stability against pH stress; (**d**) vB_PaeP_QSZH stability against pH stress; (**e**) vB_PaeP_YQZQ stability against pH stress.

The pH stability for the five phages were overall similar, and potency remained consistently high over a pH range of 3–11 within two hours. The two myoviruses, vB_ PaeM_QDB and vB_PaeM_ZGC, were inactivated at pH 2 and 12, as were the podoviruses vB_PaeP_YZ2 and vB_PaeP_QSZH. In contrast, vB_PaeP_ YQZQ exhibited exceptional tolerance to alkaline conditions and 39% of the phage remained viable at pH 12 (Figure 4).

### 3.4. Genomic Characterization

**Figure 5:**
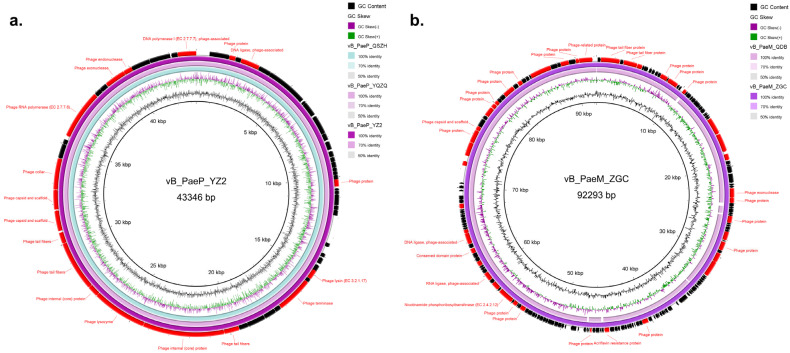
Genomic circle maps of the five phages in this study. (**a**) *Phikmvvirus* phages vB_PaeP_QSZH, vB_PaeP_YQZQ and vB_PaeP_YZ2 were compared using vB_PaeP_YZ2 as a reference. (**b**) *Pakpunavirus* phages vB_PaeM_QDB and vB_PaeM_ZGC were compared using vB_PaeM_ZGC as a reference.

We obtained the full genome sequences of these phages, and none possessed drug resistance genes and virulence factors, suggesting that they would be safe for clinical or production applications. All three *Phikmvvirus* phages possessed 43,346 bp genomes, which were highly similar (almost 100.0%) (Figure 5a). However, the genome sequences of these three phages showed some nucleotide differences (Figure S2), and the G+C content of the three phages vB_PaeP_YZ2, vB_PaeP_QSZH and vB_PaeP_YQZQ was 62.28%, 62.24% and 62.25%, respectively. The genomes of the above three phages harbored 57, 56 and 57 coding sequences (CDSs), with 4, 3 and 3 annotated as hypothetical protein, respectively. The annotated genes of these three phages included terminase large subunits and nucleic acid endo- and exo-nucleases as well as genes responsible for DNA replication, transcription, packaging, repair and phage structure. All three phages encoded holin–lysin systems.

Both the *Pakpunavirus* phages vB_PaeM_QDB and vB_PaeM_ZGC had a circular dsDNA genome, composed of 92,509 bp and 92,293 bp, respectively, and the two phages shared 97.4% similarity and 98.0% coverage (Figure 5b). Phage vB_PaeM_QDB encoded 174 CDSs, including 42 known functions, and vB_PaeM_ZGC possessed 173 CDSs, of which 42 had known functions (Figure 5b). In addition, the annotated genes of these two phages included phage terminase large subunits, conserved structural domain proteins, endo- and exo-nucleases, phosphohydrolases and proteins involved in DNA replication, transcription, packaging, repair and phage structure.

### 3.5. Phylogenetic Tree Analysis

**Figure 6:**
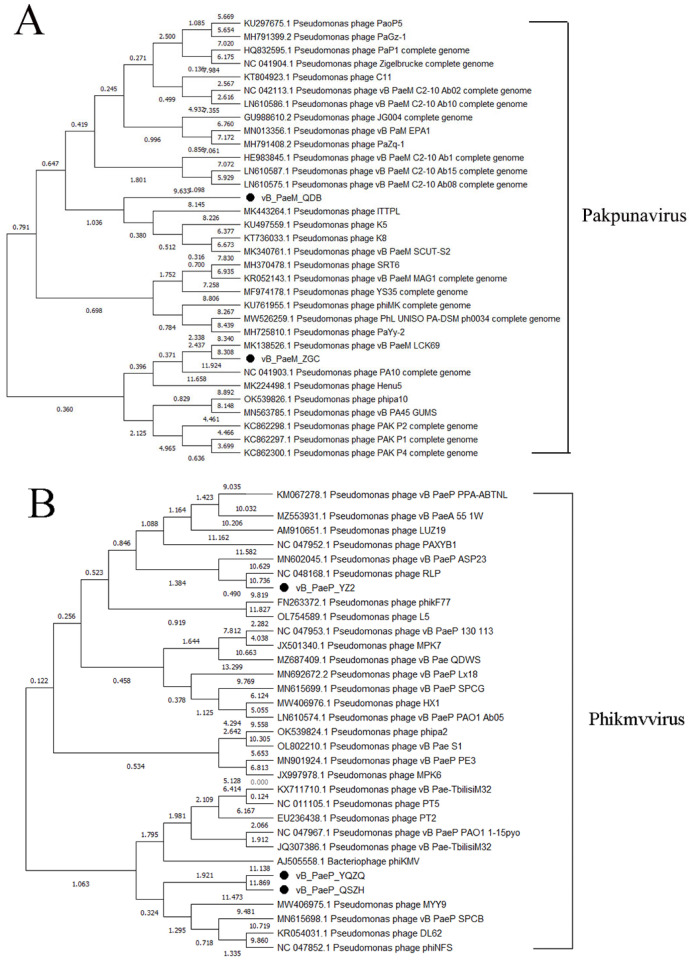
Phylogenetic trees of *P. aeruginosa* phages identified in this study and the NCBI database. (**A**) The phylogenetic relationship of *Pakpunavirus* phages in this study (labeled with ●) and related phages from NCBI. (**B**) The phylogenetic relationship of *Phikmvvirus* phages in this study (labeled with ●) and related phages from NCBI.

Based on the phage whole-genome analysis, we selected 31 phages in NCBI exhibiting high similarity (>93% identity) to our *Pakpunavirus* phages to generate a phylogenetic tree. The phylogenetic tree indicated that our *Pakpunavirus* phages vB_PaeM_QDB and vB_PaeM_ZGC were classified within *Pakpunavirus P. aeruginosa* phages. vB_PaeM_QDB demonstrated high homology with ITTPL, K5, K8 and vB_PaeM_SCUT-S2, while vB_PaeM_ZGC exhibited greater homology with vB_PaeM_LCK69 (Figure 6A).

Additionally, we selected 29 *Phikmvvirus* phages that were highly similar (>94% identiy) to our three *Phikmvvirus* phages analyzed in this study. The phylogenetic tree revealed that phages vB_PaeP_YZ2, vB_PaeP_QSZH and vB_PaeP_QSZH were clustered with a *P. aeruginosa* phage belonging to the genus *Phikmvvirus*. vB_PaeP_YZ2 displayed high homology to RLP, whereas vB_PaeP_QSZH and vB_PaeP_QSZH showed higher homology with MYY9, vB_PaeP_SPCB, DL62 and phiNFS (Figure 6B).

### 3.6. Spray Disinfection Experiment

**Figure 7:**
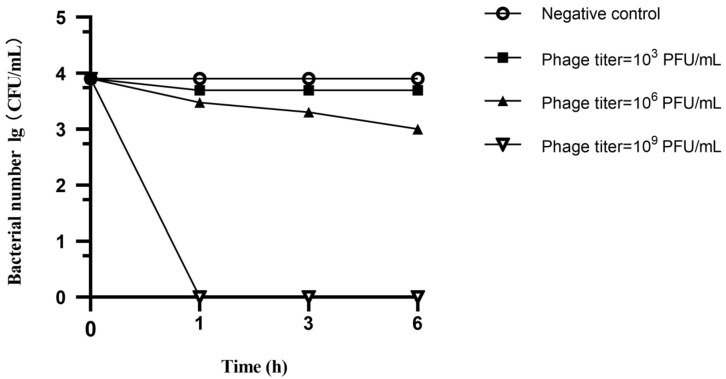
Number of colonies after spray disinfection using phage cocktails with different phage titers.

The spray disinfection test demonstrated an inhibitory effect on the growth of the six CRPA strains. Specifically, the growth of colonies following treatment with the 10^3^ PFU/mL phage cocktail was only slightly inhibited, and the differences in colony counts after 1, 3 and 6 h were not statistically significant. In contrast, treatment with the 10^6^ PFU/mL phage cocktail resulted in significant inhibition of colony growth with the number of colonies decreasing from 0.9 × 10^4^ CFU to 1 × 10^3^ CFU over the 6 h spray treatment. Remarkably, after treatment with the 10^9^ PFU/mL phage cocktail, the bacterial number dropped below detectable levels within 1 h, and this highly effective bactericidal action persisted for 6 h without any observed rebound (Figure 7).

**Figure 8:**
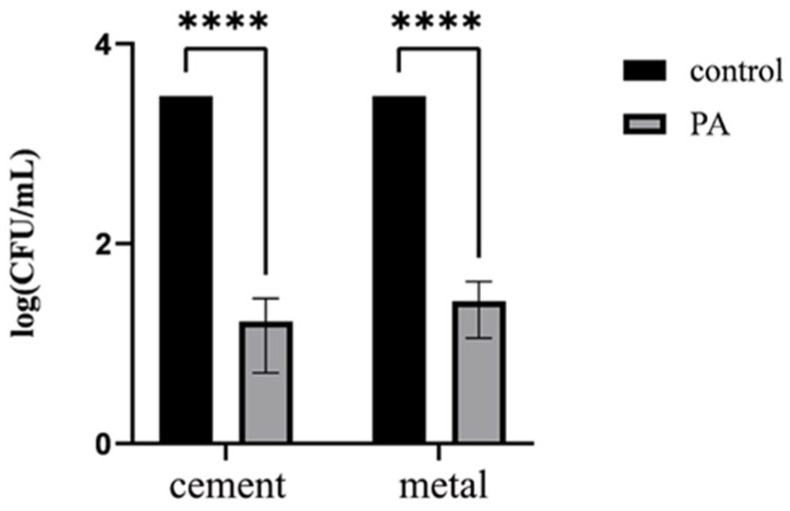
Spray disinfection on cement and metal surfaces using phages. **** indicates *p* < 0.0001.

The disinfection effect is represented in Figure 8, and the CRPA concentration of the negative control group was 3 × 10^3^ CFU/mL. The concentrations of CRPA on the cement surface in the phage treatment group in the three replicates were 1 × 10^1^, 1 × 10^1^ and 3 × 10^1^ CFU/mL, respectively. The concentrations of CRPA on the metal surface were 4 × 10^1^, 3 × 10^1^ and 1 × 10^1^ CFU/mL in the phage treatment group. The concentration of CRPA in the phage treatment group was significantly lower than that in the negative control group (*p* < 0.05), which proved that the phage spray had a significant disinfection effect on the contamination of CRPA from humans and animals on the surface of cement and metal.

## 4. Discussion

The continued global rise in CRPA and the scarcity of new antimicrobial drugs have created an urgent need for alternative treatment strategies. Phage therapy has emerged as a promising approach for controlling *P. aeruginosa* infections [30]. Phages are the most abundant biological entities on Earth, serve as ideal antimicrobial agents, and were employed against bacterial pathogens in early biotechnological applications shortly after their discovery. However, despite phage diversity, the number of available phage strains remains relatively limited. Consequently, there is a growing and pressing need to isolate potent, lytic and well-characterized phages from environmental sources for therapeutic use [31]. Phages are generally regarded as safe [32], but there is a lack of established guidelines for assessing their safety prior to clinical application. Therefore, comprehensive studies that include morphological, biological and genomic analyses of the phages intended for use are essential [30]. In this study, we obtained 94 CRPA phages, and 5 representative phages were further characterized. Three podoviruses in this study exhibited a clear round plaque at the center, surrounded by a fuzzy halo, and this was consistent with previous reports for phages of this type [33]. The halo effects suggested a decreased lysis rate due to inhibition by the high density of viral particles, resulting in secondary adsorption that delayed lysis. Additionally, the five phage strains were able to lyse most of our *P. aeruginosa* test strains and exhibited high pH and temperature tolerance, indicating significant potential for application in phage cocktail therapy.

In this study, the podoviruses exhibited a higher tolerance to temperature and pH compared to the two myoviruses, and this was consistent with a previous study and indicated that tail length was associated with phage environmental stability [34,35]. Phages with short tails (pedophages) or those without tails generally exhibit greater environmental resistance, and those with long tails (mycophages) are more susceptible to disruption that can result in a loss of their antimicrobial activity [36]. We also found that all five phages possessed double-stranded DNA genomes and none contained predicted tRNA genes or genes associated with pathogenicity, toxin production or lysogenization. Consequently, these five phage strains may be suitable for potential applications in spray disinfection. Furthermore, the presence of lytic enzymes was detected in the whole genomes of all three short-tailed phages, and this corroborated the phage plaque morphology tests [37]. Most of the phages identified thus far utilize the holin–lysin system to lyse bacteria, in which holin and lytic enzymes work synergistically to target the bacterial cell wall, and we found the presence of both a perforin and a lytic enzyme gene in each strain [38]. These phages were also significantly similar to other *P. aeruginosa* phages isolated globally, suggesting a complex evolutionary relationship [39]. There are reports of an animal-derived *P. aeruginosa* phage isolated from hospital sewage [40]. It has strong lysis ability against animal-derived *P. aeruginosa* and has a wide host spectrum, but it cannot lyse *P. aeruginosa* from humans. In this study, the five representative phages isolated were able to lyse *P. aeruginosa* from different animals and humans at the same time. Therefore, they have important clinical applications, and making them into a cocktail for spray disinfection can better reduce bacterial density. Studies have also shown that phage cocktails have a higher reduction effect on *P. aeruginosa* infections [41]. The rationale for formulating a phage cocktail is strongly supported by the distinct and complementary host ranges of the five representative phages. All phages exhibited broad lytic rates (>50%) against *P. aeruginosa* from diverse sources, and most of their individual host specificity profiles were non-overlapping. Although no single phage lysed all tested CRPA strains, their combined spectrum covered most of the *P. aeruginosa* isolates, especially those from animals. This complementary host range minimizes the risk of bacterial escape mutants and ensures synergistic coverage across animal reservoirs (poultry, swine) and human clinical settings. Such non-redundant lysis profiles are essential for effective cocktail design, as they enhance the probability of eliminating heterogeneous CRPA populations in complex environments, which is a key advantage demonstrated in our spray disinfection assays on cement and metal surfaces. These five phage strains exhibited high lysis rates and were also varied in their lysis ranges, suggesting that the creation of a phage cocktail could enhance the lysis rate. Despite the proven effectiveness of phage cocktail sterilization, certain limitations remain, and these include the dosage, application interval and phage selection. Consequently, further studies will be undertaken in the future.

While phage therapy offers a promising alternative to antibiotics, the potential emergence of bacteria resistant to phages remains a critical consideration for long-term efficacy. In this study, the use of a multiphage cocktail (targeting both *Pakpunavirus and Phikmvvirus*) may have mitigated resistance development through complementary lysis mechanisms, as observed in prior studies where phage combinations reduced resistance frequency compared to monophage treatments [42,43]. Although in vitro resistance assays were not conducted here, future work should evaluate the resistance frequency of CRPA against individual phages and the cocktail. Continuous monitoring of host–pathogen coevolution, coupled with adaptive updates to phage formulations, will be essential to sustain therapeutic utility against dynamically adapting pathogens.

However, this research has also some limitations. While the spray disinfection assay demonstrated a significant reduction in CRPA on surfaces, the static nature of the box-based model limited the dynamic assessment of phage–bacteria interactions. The discrete sampling intervals could not capture real-time kinetics of bacterial lysis or potential regrowth. Future studies should incorporate time–kill assays in liquid co-culture systems with continuous monitoring over 24 h to comprehensively quantify the bactericidal dynamics and persistence of phage activity.

## 5. Conclusions

In this study, 94 CRPA-lytic phages were isolated from 122 samples, and 5 representative phages were selected for further study, including two myoviruses and three podoviruses. The five phages exhibited lysis rates exceeding 50% against *P. aeruginosa* isolates from different sources including 23 CRPA strains from clinical patients. All podoviruses contained a perforin–lyase system. All five phages had good biological properties and possessed no drug resistance genes and virulence factors, indicating that they have the potential for safe application. Bacteriophage cocktail spray disinfection experiments demonstrated the sustained and effective ability of the phages to disinfect veterinary and human clinical environments. Further research is needed to confirm their potential applications in the future.

## Figures and Tables

**Table 1:** Phages with lysis rates above 50.0% against *P. aeruginosa* strains ^†^.

Phages	Number of CRPA Strains from Different Sources Lysed by Phage (%)							Total Number of *P. aeruginosa* Strains Lysed by Phage	Total Lysis Rates Against 246 Strains (%)
	Duck	Broiler	Laying Hen	Pig	Geese	Human	Total CRPA		
vB_PaeP_QSZH	3 (50.0)	7 (63.6)	15 (71.4)	4 (80.0)	6 (85.7)	11 (47.8)	46 (63.0)	178	72.4
vB_PaeP_YQZQ	2 (33.3)	7 (63.6)	10 (47.6)	5 (100)	6 (85.7)	12 (52.2)	42 (57.5)	176	71.5
vB_PaeP_YZ2	3 (50.0)	8 (72.7)	11 (52.4)	4 (80.0)	6 (85.7)	12 (52.2)	44 (60.3)	167	67.9
vB_PaeM_QDB	0 (0.0)	4 (36.4)	10 (47.6)	3 (60.0)	7 (100)	10 (43.5)	34 (46.5)	141	57.3
vB_PaeM_ZGC	2 (33.3)	7 (63.6)	9 (42.9)	2 (40.0)	6 (85.7)	9 (39.1)	35 (47.9)	132	53.7
vB_PaeP_YZ54	1 (16.7)	5 (45.5)	10 (47.6)	3 (60.0)	5 (71.4)	9 (39.1)	33 (45.2)	164	66.7
vB_PaeP_YZ44	1 (16.7)	3 (27.3)	2 (9.5)	1 (20.0)	5 (71.4)	8 (34.8)	20 (27.4)	130	52.8
vB_PaeP_DZSCG	1 (16.7)	1 (9.1)	5 (23.8)	1 (20.0)	5 (71.4)	8 (34.8)	21 (28.8)	129	52.4
vB_PaeM_HE1	0 (0.0)	1 (9.1)	4 (19.0)	1 (20.0)	7 (100)	9 (39.1)	22 (30.1)	140	57.1
vB_PaeM_YZ5	1 (16.7)	1 (9.1)	5 (23.8)	1 (20.0)	6 (85.7)	8 (34.8)	22 (30.1)	134	54.4
vB_PaeM_YYZ2	1 (16.7)	1 (9.1)	3 (14.3)	2 (40.0)	6 (85.7)	7 (30.4)	20 (27.4)	136	55.3
vB_PaeP_SWH	1 (16.7)	0 (0.0)	2 (9.5)	0 (0.0)	5 (71.4)	9 (39.1)	17 (23.3)	130	52.5
vB_PaeP_XHC	0 (0.0)	1 (9.1)	6 (28.6)	1 (20.0)	4 (57.1)	8 (34.8)	20 (27.4)	133	53.9

^†^ Tests were carried out using 73 CRPA and 173 meropenem-non-resistant *P. aeruginosa* strains. The percentages in the parentheses are the lysis rates of the phages against CRPA from different sources.

## Data Availability

The raw data supporting the conclusions of this article will be made available by the authors on request.

## Supplementary Materials

The following supporting information can be downloaded at https://www.mdpi.com/article/10.3390/microorganisms13071587/s1, Figure S1: Phage spot morphology of the five phages. Figure S2: Representative genomic regions showing nucleotide differences (A) between vB_PaeP_QSZH and vB_PaeP_YQZQ, vB_PaeP_QSZH as the reference genome; (B) between vB_PaeP_YZ2 and vB_PaeP_QSZH, vB_PaeP_YZ2 as the reference genome; (C) between vB_PaeP_YZ2 and vB_PaeP_YQZQ, vB_PaeP_YZ2 as the reference genome. Table S1: Lysis rate of the five phages against six CRPA strains in disinfection test.
