# Supplementary material for: Characterization, Genomic Analysis and Application of Five Lytic Phages Against Carbapenem-Resistant Pseudomonas aeruginosa
Source: Microorganisms. 2025 Jul 5;13(7):1587. doi: 10.3390/microorganisms13071587 (PMC12298563; doi:10.3390/microorganisms13071587)
Supplement: Supplementary file 1 [file microorganisms-13-01587-s001.zip › microorganisms-3636424-supplementary.pdf]

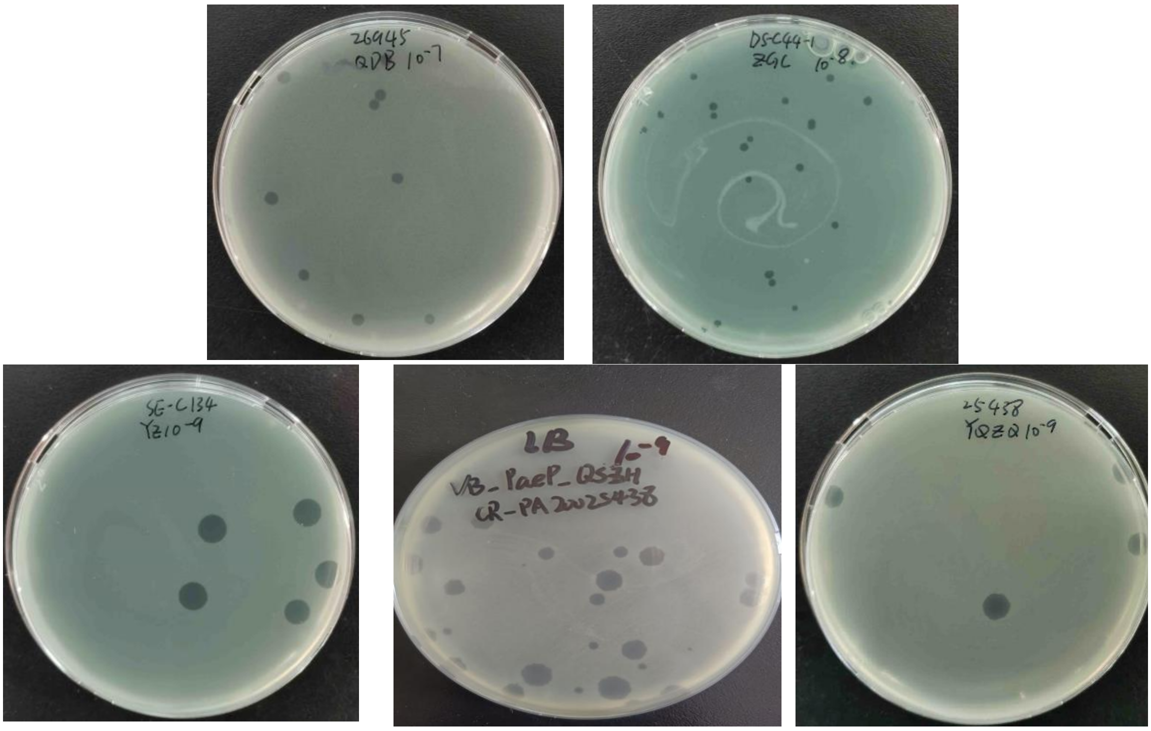

Figure S1. Phage spot morphology of the five phages

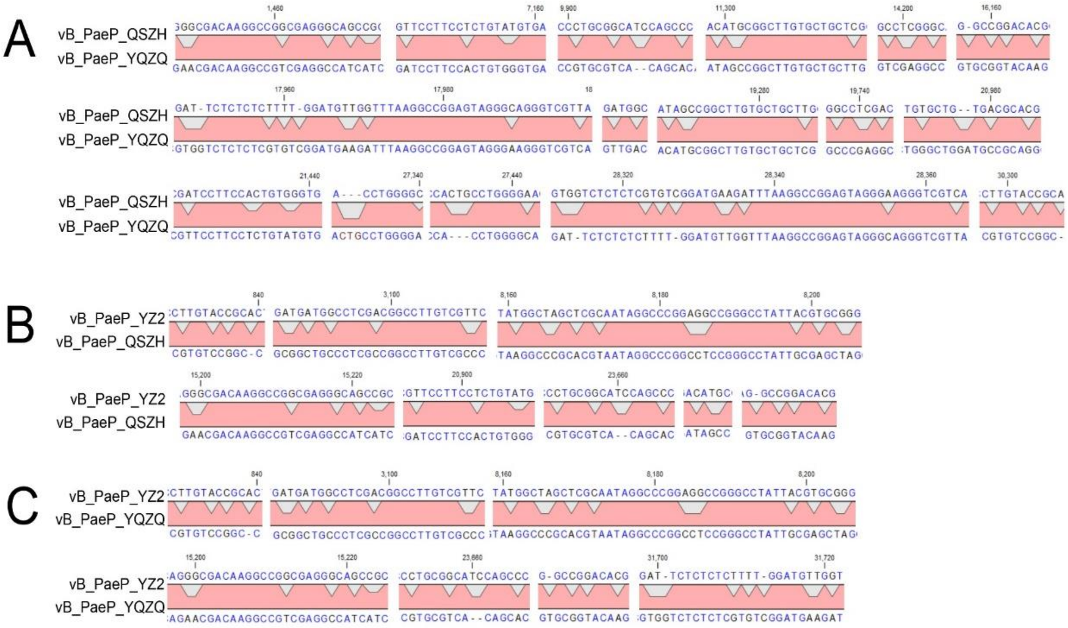

Figure S2. Representative genomic regions showing nucleotide differences (A) between vB_PaeP_QSZH and vB_PaeP_YQZQ, vB_PaeP_QSZH as the reference genome; (B) between vB_PaeP_YZ2 and vB_PaeP_QSZH, vB_PaeP_YZ2 as the reference genome; (C) between vB_PaeP_YZ2 and vB_PaeP_YQZQ, vB_PaeP_YZ2 as the reference genome.

Table S1 Lysis rate of the five phages against six CRPA strains in disinfection test

CRPA strains	Orgins of the CRPA	Phages				
		vB_PaeM_QDB	vB_PaeM_ZGC	vB_PaeP_YZ2	vB_PaeP_QSZH	vB_PaeP_YQZQ
CD4-4-1	Duck			+	+	+
JMA210PA	layer	+	+		+	
SE-C44-1	broiler			+	+	+
SE-P45-2	Swine	+		+	+	+
G1-7-1	Goose	+		+	+	+
CR-PA20026836	Patient		+		+	+
